# What have we learned from immunotherapy? Report from the 3rd and 4th meetings of the Campania Society of Oncology Immunotherapy (SCITO)

**DOI:** 10.1186/s40425-016-0144-y

**Published:** 2016-07-19

**Authors:** Paolo Antonio Ascierto, Giacomo Cartenì, Cesare Gridelli, Sandro Pignata, Antonio Pinto, Carmen Criscitiello, Luigi Buonaguro, Stefano Pepe, Roberto Mabilia, Vincenzo Montesarchio, Bruno Daniele, Sabino De Placido

**Affiliations:** Istituto Nazionale Tumori Fondazione G. Pascale – Unit of Melanoma, Cancer Immunotherapy and Innovative Therapy, Via Mariano Semmola, 80131 Naples, Italy; Dipartimento di Oncopneumoematologia, Unit of Medical Oncology, A.O.R.N. “A. Cardarelli”, Naples, Italy; Division of Medical Oncology, “S. G. Moscati” Hospital, Avellino, Italy; Department of Urology and Gynecology, Istituto Nazionale Tumori Fondazione “G. Pascale”, Via Mariano Semmola, 80131 Naples, Italy; Department of Hematology and Stem Cells Transplantation, Istituto Nazionale Tumori Fondazione “G. Pascale”, Via Mariano Semmola, 80131 Naples, Italy; Division of Early Drug Development for Innovative Therapies, Istituto Europeo di Oncologia, Milan, Italy; Department Experimental Oncology, Lab of Molecular Biology & Viral Oncology, Istituto Nazionale Tumori Fondazione “G. Pascale”, Via Mariano Semmola, 80131 Naples, Italy; Department of Medicine and Surgery, University of Salerno, Baronissi, Salerno, Italy; Unit of Oncology, P.O. “Rizzoli”of Ischia, Ischia, NA Italy; Unit of Oncology, A.O.R.N. dei COLLI “Ospedali Monaldi-Cotugno-CTO”, Naples, Italy; Department of Oncology, A.O. “G. Rummo”, Benevento, Italy; Department of Molecular and Clinical Endocrinology and Oncology, University “Federico II”, Naples, Italy

**Keywords:** Non-small-cell lung cancer, Breast cancer, Ovarian cancer, Gastrointestinal cancer, Hepatocellular carcinoma, head and neck cancer, Hodgkin’s lymphoma, Immunotherapy, Tumor-infiltrating lymphocytes

## Abstract

Treatment strategies that target the immune system provide the opportunity for antitumor activity across multiple cancer types, regardless of mutational status or tumor histology. While many of the initial advances in immunotherapy have been in melanoma, the focus has now broadened to include many other solid as well as hematological cancers. Different immunotherapeutic approaches are being evaluated across tumor types and their various novel mechanisms of action and safety profiles offer the potential for a variety of combination regimens. Ongoing and planned investigation of these immunotherapies, alone and in combination, represents the start of a new chapter in our treatment of cancer and offers the hope of better outcomes for patients with a wide range of cancers. Recent advances in the use of immune-based approaches to treat non-small-cell lung cancer, breast cancer, ovarian cancer, gastrointestinal cancer, hepatocellular carcinoma, head and neck cancer and lymphoma were discussed at the 2015 Spring and Winter meetings of the Campania Society of Oncology Immunotherapy (SCITO) and are reported here.

## Background

Both innate and adaptive T cell-mediated immunity arms are believed to play coordinated roles in cancer immune surveillance. T lymphocytes have a critical role in cancer development, with density of tumor-infiltrating T lymphocytes (TILs) being prognostic for improved outcomes in various cancers, although evidence concerning their antigen specificity and protective mechanisms of action is limited [1, 2]. A conceptual model in which an adaptive T cell response composed of both cytotoxic CD8+ T cells (CTLs) and CD4+ Th1 cells control cancer progression has been postulated, involving cytokine production and the expansion and activation of cytotoxic CD8+ T cells [3]. Immunotherapeutic approaches in cancer have mostly attempted to harness this adaptive T cell response.

The immune system, and in particular T lymphocyte activity, is regulated by a balance of co-stimulatory and co-inhibitory signals known as immune checkpoints. Normally, these act to prevent autoimmunity and damage to peripheral tissues during immune responses to infection. However, during tumour development, cancer cells can utilize immune checkpoint proteins to suppress and evade immune attack, resulting in unchecked tumor progression. Cytotoxic T-lymphocyte-associated protein-4 (CTLA-4) and programmed cell death protein 1 (PD-1) are checkpoint molecules that down-regulate T-cell activation pathways, thereby promoting tumour growth and proliferation. Inhibition of CTLA-4 and PD-1 binding to their ligands enhances T-cell activation and proliferation, leading to tumor infiltration by T-cells and tumor regression [4].

Recent advances in the use of immune-based approaches to treat cancer have led to an increased effort to assess their potential across a wider range of solid and hematological tumor types, including those for which current treatment options are limited. Developments in the use of immunotherapies in several cancers were discussed at the 2015 Spring and Winter meetings of the Campania Society of Oncology Immunotherapy (SCITO) and are reported here.

## Non-small-cell lung cancer

Increased understanding of immune evasion strategies has resulted in the development of novel immunotherapies, including several agents that target PD-1 pathway. Both anti-PD-1 and PD-L1 compounds are currently in active clinical development for the treatment of non-small-cell lung cancer (NSCLC) (Table 1).

**Table 1:** Overview of PD-L1 and PD-1 inhibitors currently in development

Compound	Lead company	Antibody type	Affinity/*K*_2_	Selected tumour types assessed	Biomarker status
Anti-PD-L1					
Atezolizumab (MPDL3280A)	Roche	Engineered IgG1 (no ADCC)	0.4 nM	NSCLC: ORR: 21 %; 24-week PFS rate: 42 % [9] Median OS: 11.4 months (vs 9.5 with docetaxel) [10]	PD-L1 tested with SP142 mAb clone, with an automated system, and evaluated on both tumoral and immune-infiltrate cells, with a cut-off of ≥5 % and ≥10 % Biomarker status still experimental
Durvalumab (MEDI4736)	AstraZeneca	Modified IgG1 (no ADCC)	NA	NSCLC: 12-week DCR: 41 %; ORR: 16 % [8] HNSCC: ORR: 12 % (25 % in PD-L1+ patients); 24-week DCR: 16 % (25 % in PD-L1+ patients) [43] Currently being assessed in first-line recurrent or metastatic HNSCC in combination with tremelimumab [44]. Also being assessed as monotherapy or in combination with tremelimumab in bladder, gastric, pancreatic, HCC and blood cancers.	PD-L1 tested with SP263 mAb clone, with an automated system, on tumoral cells, with a cut-off of ≥25 % Biomarker status still experimental
BMS-936559	Bristol-Myers Squibb	IgG4 (humanised)	NA	Being assessed in NSCLC, melanoma, and renal-cell cancer	PD-L1 tested with 28–8 mAb clone, with an automated system, on tumoral cells, with a cut-off of ≥5 % Biomarker status still experimental
Anti-PD-1					
Nivolumab	Bristol-Myers Squibb	IgG4	2.6 nM	Approved in previously-treated advanced squamous or non-squamous NSCLC and RCC, metastatic melanoma, and HL that has relapsed or progressed after HSCT and post-transplantation brentuximab vedotin. Platinum-resistant ovarian cancer: ORR: 23 %; DCR: 54 % [25]. Advanced HCC: CR: 2/39 (5 %); PR 7/39 (18 %); 6-month OS: 72 % [32] Heavily pretreated RR-HL: ORR: 87 %; CR: 22 %. Mmedian DOR and median PFS not yet reached at median follow up of 101 weeks [53]. RR-DLBCL: ORR: 36 %; median overall DOR of 22 weeks [62]. RR-FCL: ORR: 40 %. At a median follow up of 91 weeks, the median DOR for responding patients was not yet reached [62].	PD-L1 tested with 28–8 mAb clone, with an automated system, on tumoral cells, with a cut-off of ≥5 % Biomarker status: No testing required
Pembrolizumab	Merck & Co	IgG4 (humanised)	29 pM	Approved in previously-treated advanced squamous or non-squamous NSCLC and metastatic melanoma. Ovarian cancer: ORR: 11.5 %, 23.1 % of patients had evidence of tumor reduction; DCR: 34.6 % [27]. Esophageal cancer: ORR : 23 % (*n* = 5); best response was SD in 18 % (*n* = 4) and PD in 59 % (*n* = 13) [30]. Advanced gastric cancer: ORR: 22 % (95 % CI: 10–39) by central review and 33 % (95 % CI: 19–50) by investigator review. Median time to response was 8 weeks (range 7–16); median DOR: 24 weeks; 6-month PFS: 24 % and 6-month OS: 69 % [31]. HNSCC: ORR (confirmed and unconfirmed): 18.2 % (95 % CI: 11.1–27.2) with 18 partial responses and 31 with SD [39}. Recurrent/metastatic nasopharyngeal carcinoma: CR: 1/27; PR: 6/27; SD 14/27; The best ORR (confirmed and unconfirmed): 25.9 % (95 % CI, 11.1–46.3) [42]. RR-HL: ORR: 65 %; CR: 16 %; median PFS at 24 weeks: 69 % with a median DOR ≥24 weeks in 71 % of patients who achieved complete or partial response [54, 55].	PD-L1 tested with 22C3 mAb clone, with an automated system, on tumoral cells, with a cut-off of ≥1 % and ≥50 % (strong positive). Biomarker status testing required (US)
AMP-224	GlaxoSmithKline	PD-L2 IgG1 Fc fusion	NA	Being assessed in metastatic colorectal cancer in combination with stereotactic body radiation therapy.	
Other^a^					
Pidilizumab	Medivation	IgG1 (humanised)	NA	Relapsed/refractory DLBCL: CR:34 %; ORR: 51 % in patients with measurable disease after transplant. In the whole cohort of patients, 16-month PFS from first treatment: 72 %; OS >80 % [60] Relapsed/refractory FCL in combination with rituximab: ORR: 66 %; CR: 52 %;. Median PFS for all patients:18.8 months, and not reached, at time of analysis, for the 19 patients with CR/PR	NR

*ADCC* antibody-dependent cell-mediated cytotoxicity, *CR* complete response, *DCR* disease control rate, *DLBCL* diffuse large B cell lymphoma, *DOR* duration of response, *FCL* follicular lymphoma, *HCC* hepatocellular carcinoma, *HL* Hodgkin’s lymphoma, *HNSCC* head and neck squamous cell carcinoma, *HSCT* hematopoietic stem cell transplantation, *mAb* monoclonal antibody, *NA* not available, *NSCLC* non-small cell lung cancer, *ORR* overall response rate, *OS* overall survival, *PD* progressive disease, *PFS* progression-free survival, *PR* partial response, *RCC* renal cell carcinoma, *R/R* relapsed/refractory, *SD* stable disease

^a^Pidilizumab was initially believed to have been an anti-PD-1 agent; however, it has recently been stated by Medivation that this is not the case although its exact mechanism of action has not been revealed

Anti-PD-1 agents include pembrolizumab and nivolumab, both of which have been approved by the US Food and Drug Administration (FDA) for the treatment of squamous or non-squamous NSCLC. As part of the KEYNOTE-001 trial, 495 patients with previously treated and treatment-naïve advanced NSCLC were treated with pembrolizumab 2 mg/kg every three weeks or 10 mg/kg every two weeks with a median duration of follow-up of 10.9 months [5]. Overall response rate (ORR) was 19.4 % and median duration of response (DOR) was 12.5 months. Median progression-free survival (PFS) was 3.7 months and median overall survival (OS) was 12.0 months. In 204 patients evaluable by an immunohistochemistry (IHC) clinical trial assay, ORR was 45.2 % in those with membranous PD-L1 expression in ≥50 % of tumor cells (proportion score [PS] ≥50 %), compared to 16.5 % in patients with PS 1–49 and 10.7 % in patients with PS < 1 %. Among patients with a PS ≥ 50, median PFS was 6.3 months; median OS was not reached. The relationship between ORR and PD-L1 expression was observed in both previously treated and treatment-naïve patients. Pembrolizumab is being further investigated versus docetaxel in pre-treated NSCLC patients with PD-L1 ≥ 1 %, showing increased effectiveness both in terms of ORR and OS in patients with PD-L1 ≥ 50 % (KEYNOTE-010; NCT01905657) [6], versus standard of care in treatment-naïve NSCLC patients with PD-L1 ≥ 50 % (KEYNOTE-024; NCT02142738) and versus placebo in early-stage NSCLC after resection and standard adjuvant therapy (PEARLS; KEYNOTE-091; NCT02504372).

Nivolumab has also been shown to be effective and well tolerated in patients with non-squamous NSCLC in phase II/III studies [7, 8]. In addition, in the CheckMate 012 multi-arm phase I study of nivolumab, platinum doublet plus nivolumab was promising with an ORR of 33–50 %, 24-week PFS of 36–71 % and 1-year OS of 59–87 % [9]. Similarly, nivolumab was effective compared with docetaxel in patients with advanced non-squamous NSCLC who had progressed during or after platinum-based doublet chemotherapy [10]; median OS was 12.2 months with nivolumab (*n* = 292) versus 9.4 months with docetaxel (*n* = 290) and the 1-year OS rate was 51 % versus 39 %. ORR was 19 % with nivolumab versus 12 % with docetaxel and median DOR was 17.2 versus 5.6 months. Almost one-quarter (24 %) of patients on nivolumab were treated beyond RECIST v1.1-defined progression and a non-conventional benefit was observed in 16 patients (not included in best overall response). Nivolumab was associated with greater efficacy than docetaxel across all endpoints in subgroups defined according to pre-specified levels of tumor-membrane PD-1 expression (≥1 %, ≥5 %, and ≥10 %). Nivolumab has also shown improved efficacy versus docetaxel in 272 patients with advanced squamous-cell NSCLC [11]. Median OS was 9.2 months with nivolumab versus 6.0 months with docetaxel; risk of death was 41 % lower with nivolumab (hazard ratio [HR] 0.59; 95 % CI: 0.44–0.79; *p* < 0.001). At 1 year, the OS rate was 42 % with nivolumab versus 24 % with docetaxel. ORR was independent of PD-L1 expression and consistently higher for nivolumab versus docetaxel.

The anti-PD-L1 agent durvalumab (MEDI4736) has also shown encouraging antitumor activity and a manageable safety profile in patients with NSCLC. In a phase I trial of patients with various solid tumor types, disease control rate (DCR) at 12 weeks was 41 % and ORR was 16 % among 162 evaluable patients with NSCLC, with activity observed in both squamous and non-squamous histologies [12]. ORR was higher in PD-L1+ than PD-L1− patients (25 % versus 10 %). Durvalumab is now being assessed as sequential therapy in patients with locally advanced unresectable stage III NSCLC who have not progressed following platinum-based chemoradiation therapy (PACIFIC trial, NCT02125461). Another PD-L1 inhibitor in development is atezolizumab (MPDL3280A). In a phase I dose-escalation and expansion study involving 88 patients, ORR was 21 % and 24-week PFS rate was 42 % [13]. PD-L1 expression appeared to be predictive for clinical benefit. No maximum tolerated dose, dose-limiting toxicities or treatment-related deaths were observed, with the majority of adverse events (AEs) grade 1–2. In interim analysis from the randomized, phase II POPLAR trial of previously treated NSCLC patients, median OS was 11.4 months with atezolizumab versus 9.5 months with docetaxel (HR 0.78). Improved efficacy was observed with increasing PD-L1 expression; patients with the lowest PD-L1 did not appear to benefit from atezolizumab treatment [14]. Fewer patients receiving atezolizumab experienced grade ≥3 AEs and there were no unexpected toxicities. Atezolizumab is being further assessed in PD-L1+ locally advanced or metastatic NSCLC patients (BIRCH phase II study; NCT02031458) and versus docetaxel as second- or third-line treatment in locally advanced or metastatic NSCLC patients (OAK phase III study; NCT02008227).

## Breast cancer

TILs have been consistently documented in breast cancer (BC) and have been associated with prognosis. In a seminal paper published in 1992, Aaltoma and colleagues reported that lymphocytic infiltration was associated with a good prognosis, but only among rapidly proliferating tumors [15]. Similarly, the presence of TILs is observed in some BCs and has been reported to be a potential prognostic and predictive marker in some disease types, especially triple-negative (TNBC) and HER2+ BC [16, 17].

Retrospective studies from (neo)-adjuvant trials have evaluated whether TILs could identify patients with a specific outcome. Denkert et al. have shown that TIL+ tumors present a good outcome as opposed to their negative counterparts [18, 19]. Based on this observation, the ability of TILs to quantify the residual risk of relapse after adjuvant treatment has been evaluated in two retrospective analyses from randomized trials [17, 20]. More recently, the association between TILs and pathological complete response (pCR) has been further evaluated by Denkert and colleagues [19, 21]. In the GeparSixto trial, increased pCR rates were observed in TNBC and HER2+ BC with high-TIL, compared with low-TIL tumors [19]. In the NeoALTTO trial that randomized 455 women with HER2+ early-stage BC to receive either trastuzumab or lapatinib or both agents followed by the addition of weekly paclitaxel and three cycles of FEC after surgery, the presence of TILs at diagnosis was an independent prognostic marker for pCR and event-free survival [21].

**Fig. 1:**
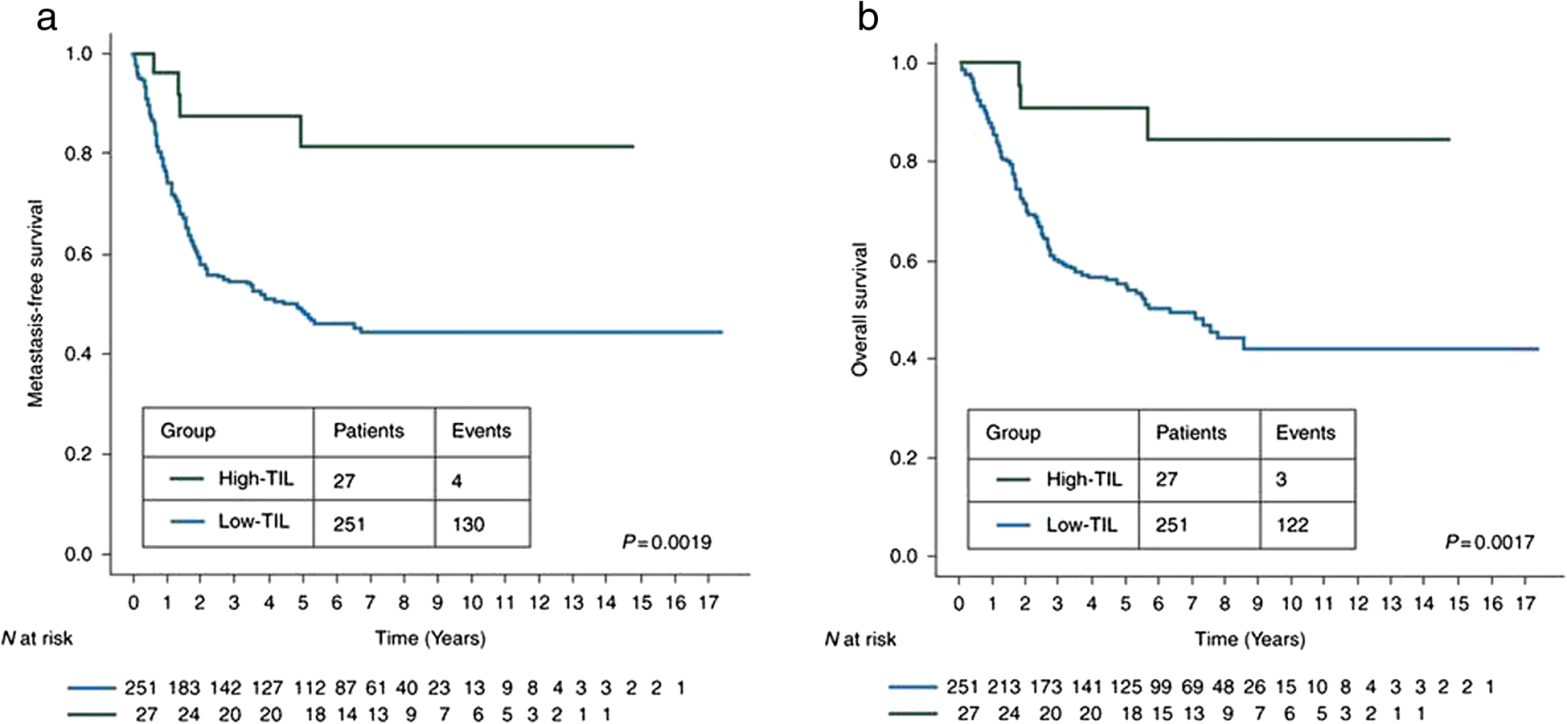
Prognostic value of tumor-infiltrating lymphocytes on residual disease after neo-adjuvant chemotherapy for triple-negative breast cancer. [22] Estimated Kaplan–Meyer curves of metastasis-free survival **a** and overall survival **b** for all patients

In a retrospective study of 304 patients with TNBC and residual disease after neoadjuvant chemotherapy, both intratumoral and stromal TILs at surgery were associated with better prognosis, especially in patients with large tumor burden (Fig. 1) [22]. Interestingly, in this study 85 % of the samples with high-TIL after neoadjuvant chemotherapy were low-TIL on core biopsies pre-neoadjuvant chemotherapy. Also, the prognostic value of TILs in TNBC has been recently confirmed by two phase III adjuvant trials [23].

Moreover, in a pooled analysis of six clinical trials involving 991 patients with TNBC treated with anthracycline-based chemotherapy, each 10 % increase in stromal TILs was associated with a 14 % relative reduction in invasive disease-free survival (IDFS) events (HR = 0.86, 95 % 0.80–0.93, *p* < 0.0001) and a 17 % relative reduction in deaths (HR = 0.83, 95 % CI 0.76–0.91, *p* = 0.0001) [24]. In a multivariate analysis adjusted for age, nodal status, tumor size and chemotherapy regimen, the HR for each 10 % increase in stromal lymphocytic infiltration was 0.86 (0.76–0.92) for IDFS events and 0.84 (0.76–0.92) for death. Moreover, stromal TILs added significant independent prognostic information for both IDFS (chi2 = 17.9; *p* < 0.0001 and OS (chi2 = 16.7; *p* < 0.0001).

TILs may also be predictive of a better response to treatment. In the BIG 02–98 trial, only in the HER2-positive BC subgroup was there evidence of a heterogeneous treatment response according to the percentage of TILs. In this subgroup, patients with high-TIL showed a higher benefit to an immunogenic chemotherapy, such as anthracyclines, as compared to those with low-TIL tumors [20].

In the FinHER trial, 232 patients with HER2+ disease were randomized to nine weeks of chemotherapy with or without trastuzumab [17]. Each 10 % increase in TILs was significantly associated with decreased distant recurrence in patients receiving trastuzumab. However, exploratory analyses from a subset of HER2+ patients in the N9831 study indicated that stromal TILs were associated with improved recurrence-free survival in patients treated with chemotherapy alone but not in patients treated with chemotherapy plus trastuzumab [25].

Evidence that a positive TIL status is associated with better outcome supports the development of immunotherapeutic strategies in patients with BC. In addition, evaluation of lymphocytic infiltrate status could help identify a subset of TIL− patients with TNBC or HER2+ BC who warrant additional therapy. Also, the ability to convert TIL− tumors into TIL+ tumors needs further investigation; such an approach might allow selecting which patients with TIL- TNBC are candidates for novel therapies in the (neo)-adjuvant setting.

## Ovarian cancer

Despite advances in combination chemotherapy regimens, current options for ovarian cancer patients are inadequate and the majority of patients will relapse. An important factor in the poor outcomes seen in patients with ovarian cancer is the lack of effective second-line treatments. As such, novel therapies need to be integrated into treatment strategies to achieve durable clinical outcomes. Immunotherapy offers a novel and promising therapeutic strategy.

Evidence for an immune role in ovarian cancer is shown by the observation that the presence of CD3+ and CD8+ TILs is associated with favorable OS in ovarian cancer. In addition, several tumor-associated antigens (TAAs) that are up-regulated in tumor tissue and ascites of ovarian cancer patients have been identified (e.g., Her2/neu, folate receptor α, p53, CA125, and members of the cancer-testis antigen family such as MAGE -A4 and NY-ESO-1). Potential immunotherapeutic approaches in ovarian cancer include monoclonal antibodies, immune checkpoint inhibitors, vaccines and adoptive cell therapy (ACT).

Antibodies include catumaxomab, a trifunctional bispecific (anti-EpCAM/anti-CD3) antibody that has been shown to improve puncture-free interval in heavily pretreated patients with chemotherapy-refractory ovarian cancer and recurrent symptomatic malignant ascites [26], the anti-EGFR agents cetuximab and panitumumab [27, 28], and antibodies that target tumor-associated macrophage (e.g., anti-CCL22, anti-B7-H4, anti-CSF-1R).

Checkpoint inhibitors are also being investigated in ovarian cancer. PD-L1 expression on monocytes in the ascites and blood of patients with ovarian cancer correlates with poor clinical outcome and cytotoxicity assays have revealed that PD-L1 overexpression on murine ovarian cancer cells inhibits cytotoxic T lymphocyte (CTL) degranulation and reduces CTL-mediated tumor lysis. In the first trial of nivolumab in patients with platinum-resistant ovarian cancer (*n* = 15), treatment resulted in a response rate of 23 % and DCR of 54 % [29]. In follow-up of two patients with complete response and one with a partial response, antitumor responses were durable and continued after discontinuation of nivolumab [30]. Pembrolizumab is also being assessed in ovarian cancer. In the ongoing non-randomized, phase Ib KEYNOTE-028 trial in patients with PD-L1+ solid tumors, pembrolizumab showed antitumor activity in an interim analysis of the patient cohort with heavily pretreated metastatic ovarian cancer (*n* = 26); ORR was 11.5 %, 23.1 % of patients had evidence of tumor reduction and DCR was 34.6 % [31]. Treatment was also generally well tolerated with no discontinuations due to toxicities. The anti-PD-L1 antibody, avelumab (MSB0010718C), has also shown promising results in ovarian cancer. In a phase Ib, open-label expansion trial, four of 23 patients (17.4 %) followed-up for ≥2 months achieved an partial response, 11 (47.8 %) had stable disease, and two had >30 % tumor shrinkage after progression was reported [32]. Median PFS was 11.9 weeks and PFS rate at 24 weeks was 33.3 % (95 % CI 11.5–57.2). The safety profile was acceptable, with fatigue, nausea, and diarrhea the most commonly reported drug-related AEs, and phase III studies are ready to start in first-line and platinum-sensitive recurrence.

Another possible approach is targeting the indoleamine 2,3-dioxygenase (IDO) pathway, an important mechanism of tumor-related immunosuppression. In ovarian cancer, IDO expression is prevalent in >50 % of surgically resected tissue, correlates with a reduced number of CD8+ TILs and NK cells, promotes tumor angiogenesis and is positively associated with impaired survival in serous-type disease. The first IDO-targeted therapy is 1-methyl-tryptophan (1-MT), a small molecule inhibitor of IDO, that prolonged survival when added to paclitaxel in an IDO-overexpressing murine ovarian cancer model and that is being evaluated in phase I clinical trials in different solid tumors with encouraging results.

Vaccine strategies in ovarian cancer include Cvac, an *ex vivo* dendritic cell vaccine that has shown promise in a phase II study in 63 patients in second remission [33], an *in vivo* dendritic cell vaccine based on the MSLN-Hsp70 fusion protein, peptide vaccines, and recombinant viral vaccines that utilize genetically modified viruses as vectors for introducing TAA-encoding DNA into cells within the body e.g., PANVAC-VF. Finally, ACT, a process that involves using autologous or allogeneic antitumor lymphocytes to induce cancer regression may have a role with the activation of endogenous T-cell immunity having been induced to enhance the elimination of tumor cells and the development of tumor-specific memory responses in a mouse model of ovarian cancer. Multiple ovarian-specific tumor antigens are being used in chimeric antigen receptor (CAR) development and ACT strategies are moving towards the clinic.

As in other cancers, the use of combination immunotherapeutic strategies is likely to be important in ovarian cancer. Strategies being investigated include anti-CTLA-4 and GVAX, a granulocyte-macrophage colony-stimulating factor (GM-CSF) gene-transfected tumor cell vaccine and nivolumab and an IDO inhibitor (INCB24360). Identification of the optimal treatment combinations should translate to substantial improvements in long-term clinical benefit.

## Gastrointestinal cancer

Despite some recent improvements, the prognosis for advanced gastric and esophageal cancer remains poor, with limited treatment options in the metastatic or unresectable setting and a typical median survival of less than one year. As with many other cancer types, immune checkpoint inhibitors may provide a new therapeutic option in the treatment of gastrointestinal (GI) neoplasms.

PD-L1 is frequently overexpressed in esophageal cancer and may be associated with a poor prognosis. In a preliminary analysis of the non-randomized, phase Ib KEYNOTE-028 trial of pembrolizumab for PD-L1+ advanced solid tumors, 90 patients with esophageal cancer were screened of whom 37 (41 %) had PD-L1+ tumors [34]. In the 23 patients treated to date, ORR (confirmed and unconfirmed) was 23 % (*n* = 5); the best response was stable disease in 18 % (*n* = 4) and progressive disease in 59 % (*n* = 13). Six patients (26 %) experienced drug-related AEs, including two (9 %) with grade 3 events. There were no grade 4 events and no patients died or discontinued due to an AE.

In the KEYNOTE-012 trial, the safety and efficacy of pembrolizumab was assessed in patients with PD-L1+ advanced gastric cancer [35]. Of the 162 patients screened, 65 (40 %) were PD-L1+ and 39 were treated with a median follow-up of 8.8 months (range 6.2–12.6). ORR was 22 % (95 % CI: 10–39) by central review and 33 % (95 % CI: 19–50) by investigator review. Median time to response was 8 weeks (range 7–16), with a median DOR of 24 weeks. The 6-month PFS rate was 24 % and 6-month OS was 69 %. PD-L1 expression level was associated with ORR. Treatment was well tolerated with four patients experiencing grade 3/4 drug-related AEs. Pembrolizumab is being further evaluated alone or in combination with chemotherapy in the phase II KEYNOTE-059 study of patients with recurrent or metastatic gastric or gastroesophageal junction adenocarcinoma (NCT023354110) and as second-line therapy in the phase III KEYNOTE-061 study versus paclitaxel in patients with advanced gastric or gastroesophageal junction adenocarcinoma after progression on chemotherapy (NCT02370498).

## Hepatocellular carcinoma

The prognosis for hepatocellular carcinoma (HCC) is generally poor given the low effectiveness of available treatments and the overall 5-year survival rate is approximately 5–6 %. Immunotherapeutic interventions may represent a novel and effective approach, although only few immunotherapy trials for HCC have been conducted and results to date have been modest.

Nivolumab was assessed in a phase I/II trial in patients with advanced hepatocellular carcinoma [36]. Drug-related AEs of any grade occurred in 29 of 41 patients (71 %); 17 % of patients had grade 3/4 AEs. A dose-limiting toxicity occurred in an uninfected patient receiving nivolumab 10 mg/kg but no maximum tolerated dose was defined. Response was evaluable in 39 patients; two had a complete response (5 %) and seven had partial responses (18 %); responses were durable. OS rate at 6 months was 72 %.

Another potential option for HCC is the development of vaccines, including pulsed dendritic cell-based vaccines and peptide vaccines. The first HCC vaccine tested was based on CD8+ T-cell epitopes specific for α-fetoprotein (AFP), showing the generation of AFP-specific T-cell responses in vaccinated subjects [37]. The same group also conducted a subsequent phase I/II trial administering AFP epitopes presented by autologous dendritic cells loaded *ex vivo*; however, this only produced a transient CD8+ T-cell responses, possibly caused by the lack of CD4+ support [38]. To increase the number of tumor TAAs elicited by the vaccine, approaches using autologous dendritic cells pulsed *ex vivo* with a lysate of the autologous tumor [39] or of hepatoblastoma cell line HepG2 [40] have been evaluated in clinical trials. However, results have been unsatisfactory.

**Fig. 2:**
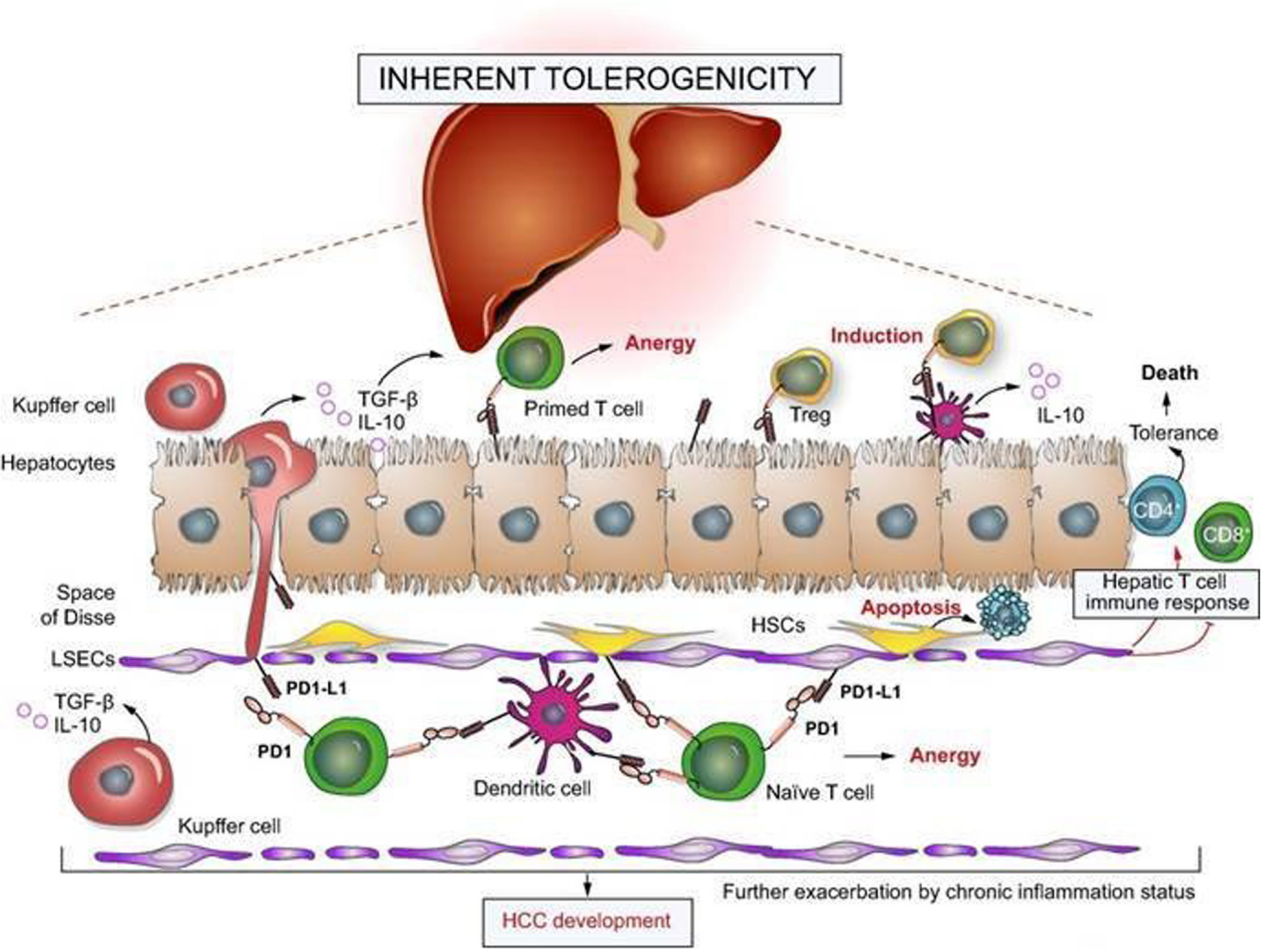
Limiting factors in immune-based approaches in hepatocellular carcinoma [f41]

One explanation for the limited success of HCC vaccines to date is that the liver is an inherently immunosuppressive microenvironment, a state that may be further exacerbated by chronic inflammation due to hepatitis infection (Fig. 2). Moreover, TAAs used in such clinical trials are not HCC-specific. In order to improve the efficacy of HCC vaccines, new and more specific TAAs and/or tumor epitopes need to be identified, both HLA class I and II restricted, aiming at inducing CD4+ as well as CD8+ activation. In addition, improved immune responses elicited by HCC vaccines may be achieved by adjuvant strategies that increase the immunogenicity of the vaccine antigen and/or counteract the immunosuppressive tumor environment. These include combination approaches with chemotherapy or loco-regional treatments (e.g., tumor ablation, transarterial chemoembolization [TACE]), the use of novel immunomodulatory adjuvants, and delivery systems that increase antigen presentation (e.g., biodegradeable nanoparticles as antigen carriers) [41].

An important initiative in the development of a vaccine for HCC is the EU-supported HEPAVAC project (www.hepavac.eu), aiming at developing a novel therapeutic HCC vaccine with multiple TAAs that are presented on the surface of primary HCC cells. This will involve on an ‘off-the-shelf’ vaccine comprising 18 newly identified MHC-I and II tumor-associated peptides (TUMAPs) naturally processed and presented on primary tumor tissues from HCC patients together with an actively personalised vaccine (APVAC) approach involving patient-specific mutated peptides. Both vaccines will be combined with a novel and potent RNA-based immunomodulator (RNAdjuvant®). Feasibility, safety and biological efficacy will be evaluated in a randomized, controlled multicentre phase I/II clinical trial, and will hopefully result in the first multi-epitope, multitarget and multi-HLA allele therapeutic cancer vaccine for this frequent and aggressive disease [42].

## Head and neck cancer

Treatment options for recurrent/metastatic head and neck squamous cell carcinoma (HNSCC) remain poor with a median OS of 10 months in the first-line setting and 6 months in previously treated patients. Human papillomavirus (HPV) is recognized as the causative agent of HNSCC in a growing subset of patients. Prominent immune escape during malignant progression is observed in HNSCC, with the PD-1/PD-L1 pathway playing an important role. The majority of HPV+ and a subset of HPV− HNSCC tumors are PD-L1+. Thus, blocking PD-1 interaction with PD-L1 or PD-L2 may reactivate immune surveillance and elicit antitumor activity.

Pembrolizumab has demonstrated antitumor activity in multiple tumor types, including HNSCC. The KEYNOTE-012 phase 1b multi-cohort study in patients with advanced solid tumors included a HNSCC expansion cohort with 132 patients irrespective of PD-L1 or HPV status who received fixed-dose pembrolizumab 200 mg every three weeks [43]. In 99 patients available for preliminary efficacy analysis, ORR (confirmed and unconfirmed) was 18.2 % (95 % CI: 11.1–27.2) with 18 partial responses and 31 with stable disease. Pembrolizumab was active in both HPV+ and HPV− patients. Drug-related AEs of any grade occurred in 47 % of patients and drug-related grade ≥3 AEs occurred in 7.6 %. In a further evaluation of immune-related gene expression patterns in 43 of these patients, the ‘Interferon-γ (IFN-γ) 0 10-gene’, ‘expanded-immune 28-gene’ and ‘de novo’ signatures showed significant associations with ORR and PFS.

In the expansion cohort of the phase Ib KEYNOTE-028 trial, the antitumor activity of pembrolizumab 10 mg/kg every two weeks is being assessed in patients with PD-L1+ recurrent/metastatic nasopharyngeal carcinoma (NPC). PD-L1 expression in NPC is upregulated by Epstein-Barr virus (EBV) induced activation of LMP1 and IFN-γ pathways and PD-1/PD-L1 expression may correlate with poor prognosis [44, 45]. Among 27 patients, one had a complete response, six had partial responses and 14 had stable disease. The best overall (confirmed and unconfirmed) response rate was 25.9 % (95 % CI, 11.1–46.3) [46]. Pembrolizumab was well tolerated with drug-related AEs observed in 70.4 % of patients. This represents the first demonstration of antitumor activity of a PD-1 inhibitor in recurrent/metastatic NPC and further investigation is planned.

Durvalumab is also being assessed in HNSCC, in an ongoing open-label, phase I/II study in multiple solid tumor types [47]. In 51 of 62 HNSCC patients evaluable for response with ≥24 weeks of follow-up, ORR was 12 % (25 % in PD-L1+ patients), and DCR at 24 weeks was 16 % (25 % in PD-L1+ patients). Drug-related AEs were observed in 60 % of patients, with fatigue, diarrhea, and nausea the most frequent. Grade ≥ 3 drug-related AEs were reported in 7 % of patients: rash (*n* = 2), and increased GGT, fatigue, and tumor inflammation (*n* = 1 for each). Durvalumab is also being assessed in combination with the CTLA-4 inhibitor tremelimumab versus standard of care for the treatment of first-line recurrent or metastatic HNSCC [48].

## Lymphomas

Application of immunotherapy to the management of lymphomas poses unique challenges and opportunities since these malignancies originate from the immune system itself [49]. Lymphomas represent the fifth most common cancer in developed countries and collectively display an age-adjusted incidence of about 23 cases per 100,000 individuals. In the Western world, the greatest majority of lymphoid malignancies arise from mature B cells that, throughout the complex pathway to generate cells producing antibodies with a high specificity and avidity, accumulate genetic and epigenetic changes incompatible with their proper function. Studies have shown that most B cell non-Hodgkin lymphomas (NHL) and Hodgkin lymphoma (HL) derive from these nonfunctional B cells escaping the apoptotic death within secondary lymphoid tissues [50]. Similar mechanisms, involving errors in the generation of a functionally active T-cell receptor have been implied in the pathogenesis of the more rare T-cell NHLs [50]. The cellular and clinical heterogeneity of lymphomas reflects the complexity of the human immune system including the intricate patterns of cellular interplay underlying the functional regulation of the immune response. Beyond the focus on the genetic changes and cell-signaling aberrations that may act as targets for a tailored therapeutic intervention across the various lymphoma subtypes, studies have recently recognized that mechanisms operated by lymphoma cells to evade antitumor immunity through the generation and maintenance of a tolerogenic tumor microenvironment can represent a further area to build newer immunotherapeutic approaches [51, 52].

Among these strategies, immune checkpoint inhibition is emerging with considerable promise in the treatment of NHL and HL. However, at variance with solid tumors, lymphomas are characterized by a more promiscuous pattern of reciprocal expression of the receptor-ligand pairs of the PD-1 pathway among tumor cells and non-malignant lymphoid cells. These expression patterns are also significantly variable across the specific lymphoma subtypes [53]. The expression of PD-L1/2 on tumor cells can be regulated by IFN-γ, IL-4 and other cytokines produced in the tumor environment typical of some lymphomas, while, in other lymphoma subtypes, the constitutive expression of PD-L1/2 on tumor cells is related to a specific acquired genetic trait. Tumor cells of some lymphoma subtypes do not directly express PD-1-L, but PD-L1+ histiocytes and other microenvironmental cells are present which contribute to the exhaustion of tumor PD-1+ infiltrating T/NK cells. As an example, while in HL and some biologically related NHL, such as the primary mediastinal B-cell lymphoma (PMBCL), tumor cells clearly overexpress PD-L1/2, in follicular cell lymphoma (FCL), the prototype of indolent NHL, malignant cells are usually negative for expression of both PD-1 ligands. In aggressive NHL, the patterns of expression of PD-1 and its ligands in tumor cells might not differ from their non-malignant counterpart cells or display a subtype-divergent picture as in the case of the germinal-center (GC) and activated B cell (ABC) variants of diffuse large B-cell lymphoma (DLBCL). Such heterogeneity depends on specific genetic features acquired by tumor cells, the variable milieu of cytokine-mediated and cell contact-dependent interactions among malignant and non-malignant cell populations within the lymphoma microenvironment or on the combination of all these factors.

## Hodgkin’s lymphoma

**Fig. 3:**
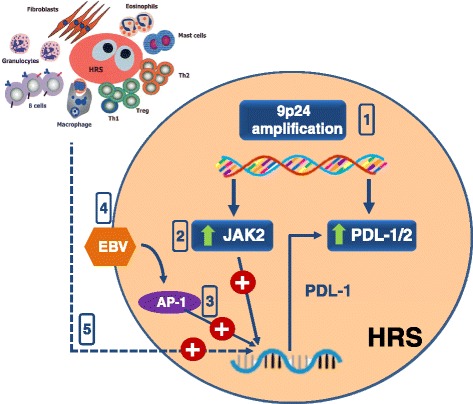
Mechanisms leading to PD1 ligands expression in Hodgkin Reed-Sternberg (HRS) cells of Hodgkin Lymphoma. Multiple mechanism are concurrently implicated in overexpression of PD1-ligands by tumor cells of Hodgkin lymphoma. 1) Amplification of 9p24 increases gene dosage for PD-L1/2 and JAK2. 2) JAK2 gene product increases transcription of the PD-L1 gene. 3) HRS cells display the constitutive activation of AP1 whivh binds to an AP-1–responsive enhancer in the PD-L1 gene. 4) The Epstein-Barr Virus (EBV)-encoded latent membrane protein 1 increases PD-L1 promoter activity, also via AP-1. 5) The network underlying tumor microenvironment formation and maintenance in Hodgkin lymphoma results in the constitutive production of cytokines known to enhance activity of the PD-L1 gene

PD-L1 and/or PD-L2 over-expression on lymphoma cells may play a critical role in immune evasion in patients with HL. The PD-L1 and PD-L2 genes are located on chromosome 9p24.1, over-amplification of which represents a specific structural alteration that occurs in some cases of HL; this increases the gene dosage of PD-1 ligands as well as their induction by JAK2 whose gene is located in the same amplified chromosomal trait [54]. As such, the PD-1 pathway and JAK2 may represent complementary therapeutic targets in HL (Fig. 3). However, tumor cells from HL cases with normal 9p24.1 copy numbers also appear to over express PD-1 ligands. The presence of an AP-1–responsive enhancer in the PD-L1 gene along with the constitutive AP-1 activation, typical of tumor cells of HL, may then depict an alternative mechanism leading to PD-1 ligand overexpression in this lymphoma [55]. Finally, the demonstration that the EBV-encoded latent membrane protein 1 increases PD-L1 promoter activity supports a third mechanism for PD-L1 overexpression in HL, since EBV-encoded proteins can be found in about 40 % of HL cases [55]. Such concurrency of mechanisms may explain why overexpression of PD1-ligands is a phenotypic trait common to almost all cases of HL. While this situation clearly accounts, from one side, for the impressive clinical efficacy of PD1-inhibitors in HL, it may hamper, from the other, the use of PD-L1/2 expression on tumor cells as a predictive biomarker for response.

The therapeutic use of PD-1 inhibitors, nivolumab and pembrolizumab, in patients with relapsed and refractory (RR) HL has been initially explored in two early-phase studies. In the expansion cohort of the phase Ib trial (CA209-039), 23 patients with heavily pretreated RR- HL were given nivolumab 3 mg/kg every two weeks until response, tumor progression or intolerable side-effects [56]. Of the enrolled patients, 78 % had progressed after autologous stem cell transplantation (ASCT) and 78 % had failed on brentuximab vedotin (BV). ORR was 87 % with a complete response rate of 22 %. At a median follow up of 101 weeks, the median DOR and median PFS were not yet reached [57]. Nivolumab was overall well-tolerated with few grade 3/4 toxicities, mostly involving blood, skin, the gastrointestinal tract and lungs. Interestingly, in the one patient that was retreated with nivolumab post-progression, a second response was achieved. Analysis of pretreatment tumor specimens from 10 patients revealed copy-number gains and increased expression for both PD-L1 and PD-L2. Within the Checkmate 205 program, the CA209-205 study in RR-HL has recently completed the accrual for the original three cohorts, involving more than 250 patients, and a further cohort is being opened to test upfront treatment with single agent nivolumab followed by a nivolumab/AVD (doxorubicin, vinblastine, dacarbazine) combination for chemo-naïve patients with advanced HL.

In the HL cohort of the KEYNOTE-013 study, 31 patients with RR-HL, who had progressed after BV and 67 % of whom had also failed ASCT, received pembrolizumab (10 mg/kg every 2 weeks) [58, 59]. The ORR was 65 % with a CR rate of 16 %. The median PFS at 24 weeks was 69 % with a median DOR ≥24 weeks in 71 % of patients who achieved complete or partial response (range: 0.14–74+ weeks). There were no AEs of grade higher than 3 and the most common treatment-related AEs were hypothyroidism (16 %), diarrhea (13 %), nausea (13 %), and pneumonitis (10 %). Based on these impressive response rates and significant response duration, a number of further trials involving PD-1- (e.g., nivolumab, pembrolizumab) and PD-L1- (durvalumab) targeted antibodies have been activated in patients with RR-HL. Furthermore, combinations of anti-PD1 antibodies with other immune checkpoints inhibitors such as ipilimumab and with BV are being currently tested in the same patient setting. The combination of PD-1 inhibitors with BV appears particularly intriguing since the sustained clinical response achieved with this latter agent, an anti-CD30 antibody conjugated with monomethyl-auristatin E, a toxic poison of the dolastin family, have been related, beyond its direct cytotoxic activity, to the stimulation of an anticancer immune response [60]. In an immunocompromised murine model, therapeutic synergies were observed when combining dolastatins with tumor antigen-specific vaccination or PD-1/PD-L1 and CTLA-4 blockade [61]. This provides a strong rationale for treatment strategies that combine BV with immune-based therapies in patients with HL.

## Non-Hodgkin lymphoma

In this group of lymphomas, the more heterogeneous pattern of PD-1 and its ligands on tumor cells may render more complex the interpretation of early clinical results obtained with some of the immune checkpoint inhibitors. One exception is represented by PMBCL, since in this aggressive NHL tumor cells often share the 9p24.1 amplification and constitutive PD-L1/2 overexpression typical of HL [54]. In this regard, the PMBCL cohort of KEYNOTE-013 study enrolled 10 heavily pretreated patients who also relapsed after or were ineligible for ASCT [62]. In nine evaluable patients, the ORR was 44 %, with one patient achieving a CR and three patients achieving a partial response. At a median follow-up of 144 days, the median DOR was not reached (1+ to 291+ days), with all four responses ongoing at the time of data cutoff. These preliminary results fully justify further studies in this unique type of NHL.

Within the aggressive lymphomas, DLBCL represents the most common form accounting for about 30 to 35 % of all NHL in the adults. Based on specific molecular signatures, two main biological subtypes with a different prognosis have been identified. In the GC-type of DLBCL, tumor cells rarely express PD-L1/2 or PD-1 and the presence of PD-1+ T cells in the microenvironment is of unusual occurrence [53, 63]. Conversely, in DLBCL of ABC-type, neoplastic cells are characterized by a more sustained expression of PD-L1/2, but not of PD-1, and an excess of PD-1+ T cells can be found in the tumor microenvironment in some cases [53, 63]. In both CG- and ABC-DLBCL, however, variable amounts of PD-L1 and/or PD-1-expressing monocytes, histiocytes, dendritic cells and T/NK cells can be found in the microenvironment, supporting the presence of PD-1-mediated interactions in the immune networks operating in these tumors [53].

In a first single-arm phase 2 study, 66 eligible patients with RR-DLBCL, were given pidilizumab (1.5 mg/kg every 42 days) in the attempt to prevent early progression in those showing overt residual disease after ASCT and to consolidate response in those who achieved a complete response after transplantation [64]. Treatment yielded to a CR rate of 34 % and ORR of 51 % among patients with measurable disease after transplant. In the whole cohort of patients a 16-month PFS from first treatment of 72 % was recorded along with an OS exceeding 80 %. These figures met the primary study endpoints and a concurrent, hypothesis-generating, immunoprofiling of circulating cells was consistent with an ‘on-target ‘*in vivo* effect of pidilizumab. No data were available as to relative frequencies of GC- versus ABC-DLBCL subtypes within the accrued patient population. Of note, it has recently been revealed that the target of pidilizumab is not, as was originally thought, PD-L1. Although its exact mechanism of action has not yet been explained, it has been stated that its administration is associated with enhanced maturation and survival of T lymphocytes, which may improve adaptive immunity, as well as activation of natural killer cells, which may improve innate immunity [65]. In the expansion cohort of the CA209-139 nivolumab study in lymphoid malignancies, 10 RR-DLBCL patients, failing after or ineligible for ASCT, were enrolled [66]. The ORR was 36 % and a complete response was obtained in two patients, with a median overall DOR of 22 weeks.

FCL, beyond representing the second most common form of NHL in the Western world (22-25 % of all cases), may be of particular interest in the setting of immune checkpoint blockade strategies. Several molecular studies have confirmed that specific ‘immune signatures’, mirroring the presence and activity of several types of immune effectors in the lymphoma microenvironment, may strongly and independently predict prognosis in this indolent lymphoma [67]. In addition, while tumor cells do not usually express PD-L1 or PD-1, the FCL microenvironment is highly enriched for PD-1 and/or PD-L1/2 immune effectors [53]. In a single-arm phase 2 study, 29 eligible patients with RR-FCL, all previously exposed to rituximab, were treated with four courses of pidilizumab (3 mg/kg every 4 weeks) followed by eight monthly optional infusions in patients showing at least a stable disease [68]. After 17 days from the first infusion of pidilizumab, patients also received four weekly doses (375 mg/m^2^) of rituximab. The combination of pidilizumab plus rituximab was very well tolerated and devoid of grade 3 and 4 adverse events. Of the enrolled patients, 19 (66 %) achieved an objective response, that was complete in 15 (52 %). The median PFS for all patients was of 18.8 months, and was not reached, at time of analysis, for the 19 patients in complete or partial response after pidilizumab plus rituximab. A concurrent immunoprofiling study, confirmed the ‘on target’ activity of the combination. The expansion cohort of the CA209-139 nivolumab study enrolled 10 patients with RR-FCL [66]. Four patients (40 %) achieved an objective response, including a complete response and four partial responses. At a median follow up of 91 weeks, the median DOR for responding patients was not yet reached. The favorable toxicity profile of nivolumab was confirmed in these patients.

These extremely promising results are prompting an increasing number of trials targeted to specific NHL subtypes to better establish activity and mechanism of action of different immune checkpoint inhibiting-agents within such a heterogeneous group of tumors. Studies are also being launched to test newer combinations with antibodies targeting CD20 and other surface structures of tumor B cells and with molecules which target the known cell-signaling aberrations of the various NHL subtypes. The outstanding results obtained in patients with RR-HL led to the accelerated approval of nivolumab in the US and to its ongoing registration in Europe, where patient-named programs with this agent are currently ongoing. Studies testing the introduction of immune checkpoint inhibitors for the upfront treatment of HL have already been launched.

## Conclusions

Treatment strategies that target the immune system provide the opportunity for antitumor activity across multiple cancer types, regardless of mutational status or tumor histology. While many of the initial advances in immunotherapy have been in melanoma, the focus has now broadened to include many other solid as well as hematological cancers. Different immunotherapeutic approaches are being evaluated across tumor types and their various novel mechanisms of action and safety profiles offer the potential for a variety of combination regimens. Ongoing and planned investigation of these immunotherapies, alone and in combination, represents the start of a new chapter in our treatment of cancer and offers the hope of better outcomes for patients with a wide range of cancers.
